# Social stigma and its relationship with quality of life in multiple sclerosis patients

**DOI:** 10.1186/s12883-023-03395-0

**Published:** 2023-11-17

**Authors:** Nader Sharifi, Vahid Kohpeima Jahromi, Razieh Zahedi, Shabnam Aram, Maryam Ahmadi

**Affiliations:** 1 Department of Public Health, Khomein University of Medical Sciences, Khomein, Iran; 2 Research Center for Social Determinants of Health, Jahrom University of Medical Sciences, Jahrom, Iran

**Keywords:** Multiple sclerosis, Social stigma, Quality of life

## Abstract

**Introduction:**

Multiple sclerosis (MS) is the most prevalent disease of the central nervous system that affects the behavioral characteristics and lifestyle of patients. This study aimed to determine the social stigma and its relationship with quality of life in people with MS referring to the Jahrom MS Association.

**Method:**

This cross-sectional study was conducted on MS patients who are members of the MS Association of Jahrom City in Fars province, southern Iran in 2022. The samples were selected by census method. The number of participants remain 223 people. The data was collected using a standard two-part demographic and Multiple Sclerosis Quality of Life-54 (MSQOL-54) questionnaire and a researcher-made 20-question Social stigma questionnaire which validity and reliability were measured. Data analysis was done in a descriptive way (frequency, percentage, mean and standard deviation), univariate regression and multivariable linear regression. Data analysis was done using SPSS version 17 and at a significance level of less than 0.05.

**Result:**

Based on univariate regression, marriage [B = 0.2, p-value = 0.004], physical health [B = 0.4, p-value < 0.001], emotional well-being [B = 0.4, p-value = 0.001], cognitive functioning [B = 0.4, p-value < 0.001], health distress [B = 0.5, p-value < 0.001] had a positive and significant relationship with patients’ quality of life. Duration of disease [B=-0.2, p-value < 0.001], physical role limitations [B=-0.4, p-value < 0.001], emotional role limitations [B=-0.5, p-value < 0.001], pain [B=-0.4, p-value < 0.001], energy [B=-0.3, p-value = 0.02], health perception [B=-0.3, p-value = 0.001], social functioning [B=-0.4, p-value < 0.001], sexual function [B=-0.3, p-value < 0.001], change in health [B=-0.3, p-value < 0.001], sexual function satisfaction [B=-0.3, p-value < 0.001] and social stigma [B=-0.3, p-value < 0.001] had a negative and significant relationship with patients’ quality of life (p < 0.05).

**Conclusion:**

The study has emphasized the relationship between the extent and severity of symptoms and disorders with the quality of life of people with MS. The results of the study showed factors such as marriage, physical health and health distress have a positive relationship and factors like duration of disease, physical role limitations, and social stigma have a negative relationship with the quality of life of people with MS. The quality of life of people with MS is more influenced by mental and psychological factors than the physical limitations of these patients.

## Introduction

Multiple sclerosis (MS) is the most prevalent disease of the central nervous system, which is caused by the breakdown of the myelin sheath and affects the behavioral characteristics and lifestyle of patients [1, 2]. Anxiety, depression, stress and fatigue are the most typical psychological symptoms of MS [3, 4]. The global prevalence of MS in 2020 is estimated at 35.9 per 100,000 people [5], while the results of a recent systematic review study indicated the prevalence of MS in Iran to be 100 per 100,000 [6]. More than 70,000 people with MS live in Iran, which ranks first in the Middle East [7]. The results of a study indicated a relatively high prevalence of the disease in Fars province and Jahrom city compared to other parts of Iran, the reason for which may be related to climatic and geographical differences, as well as racial and cultural differences [8]. People with MS experience reduced quality of life due to physical limitations and psychosocial challenges that include sexual dysfunction, problems with defecation, adverse emotional functioning, and shame [9, 10]. These patients possess a lower quality of life compared to healthy people and those suffering from other chronic diseases [11]. Studies have shown that various psycho-social factors, including feelings of helplessness and social stigma, affect the quality of life of people with MS [12, 13]. Goffman introduces social stigma as a destroyed identity that causes people to be devalued and unwanted isolation due to the characteristics that are attributed to and as a result, society does not consider these people to have the necessary sufficiency to be accepted [14, 15]. People with MS experience mild to moderate social stigma as a result of the disease [16], and this social stigma has potential consequences for their health and quality of life [17], so people with the disease discover social stigma an obstacle to their daily functioning and flexibility [18]. Socially stigmatized patients are frustrated and isolated, which reduces the use of medical services and also weakens their quality of life [19]. Social stigma in MS disease may cause increased anxiety, depression and decreased self-esteem [16]. Broersma’s study showed social stigma was extremely common in people with MS and that patients who experience less restriction and social stigma have a better quality of life [12]. Moreover, the results of Anagnostouli’s study showed there was a significant negative correlation between the intensity of social stigma and quality of life variables [16]. Considering the relatively large number of MS patients in Jahrom city, as well as relatively little research that has been done on how social stigma affects MS disease [20], Examining the quality of life and its relationship with stigma will carry out a significant role in increasing the understanding of health care providers and families on this issue; therefore, the present study was designed to reveal the effects of stigma on the quality of life for the people of the community and the patients themselves, to obtain the necessary measures to improve the quality of life of people with MS and influence the negative attitude of the society towards them. This study aimed to determine the social stigma and its relationship with quality of life in people with MS referring to the Jahrom MS Association.

Hypotheses: There is a relationship between various aspects of quality of life and stigma in people with multiple sclerosis. There is a relationship between demographic variables and quality of life in people with multiple sclerosis.

## Method

### Study design and population

This cross-sectional study was conducted on MS patients who are members of the MS Association of Jahrom City in Fars province, southern Iran in 2022.

### Sampling procedure

The samples were selected by census method and included all members of the MS Association. Due to the limitation of the number of people with MS in the city, all the research populations that met the inclusion and exclusion criteria were included in the study. To identify the samples, the names of the people were extracted from their files in the MS Association. The inclusion criteria included all people with MS who were members of the Jahrom MS Association, and the exclusion criteria included failure to complete the written consent form, critically ill patients, and inability to answer the questionnaires. The number of participants in the study remain 223 people. The stages of identifying samples and inviting people to participate in the study and the final selection of participants were done within 45 days. In addition, data collection and analysis were done in three months.

### Study instruments

The data were collected using a standard (its validity and reliability confirmed in Iran) two-part demographic (including age, gender, marital status, place of residence, age of disease onset, duration of MS disease and type of disease course) and Multiple Sclerosis Quality of Life-54 (MSQOL-54) questionnaire and a researcher-made 20-question Social stigma questionnaire. MSQOL-54 contains 54 questions, 18 of which are in 14 domains specific to MS patients and 36 are related to the general quality of life. The questions have two to seven options and are based on the Likert scale. Finally, the score of the patient’s quality of life is determined by the scores produced for the two combined domains. The combined domain of physical health of quality of life is composed of the scores of the domains of physical function (including weight 0.17), health perception (0.17), energy (0.12), physical role limitations (0.12), pain (0.11), sexual function (0.08), social function (0.12) and health distress (0.11), which is a number between zero and 100. The combined domain of mental health is also composed of the total scores of the areas of change in health (0.14), general quality of life (0.18), emotional well-being (0.29), emotional role limitations (0.24) and cognitive function (0.15), which is a number between zero and 100. For all domains, higher scores indicate better status. To use the MSQOL-54 questionnaire in Iran, using the method of translation and de-translation and measuring patients’ questions, the Persian version of the questionnaire was prepared and approved by Ghaem and Haghighi [21]. They measured and confirmed the face and content validity of this tool and declared its reliability higher than 0.7 using Cronbach’s alpha method. In the present study, the reliability of this questionnaire was evaluated, and Cronbach’s alpha values for the total questionnaire were 0.81, physical function 0.74, health perception 0.74, energy 0.8, physical role limitations 0.71, pain 0.9, sexual function 0.81, social function 0. 0.73, health distress 0.7, change in health 0.73, the general quality of life 0.81, emotional well-being 0.82, emotional role limitations 0.73 and cognitive function 0.82 were obtained.

The researcher-made Social stigma questionnaire was prepared after reviewing similar texts and articles [10, 12, 13, 17, 19] and using the opinions of experts in related fields. The questionnaire contains 20 questions about different aspects of social stigma. In this questionnaire, a five-point Likert scale (totally disagree, disagree, neither disagree nor agree, agree, totally agree) was implemented and the score of each question was between one and five and each respondent can get a minimum of 20 and a maximum of 100 points; the higher the score, the greater the social stigma. In face validity evaluation, the questionnaire was given to 20 MS patients whose conditions were similar to those eligible for the study. Items such as understanding phrases and words, the degree of appropriateness and the optimal connection of phrases with the dimensions of the questionnaire and the level of difficulty were investigated. To determine the qualitative content validity, the questionnaire was given to eight experts in health education and tool design, and the questionnaire was modified based on the criteria of grammar, use of appropriate words, appropriateness of selected dimensions and appropriate scoring. In the quantitative evaluation of content validity, two content validity ratios (CVR) and content validity index (CVI) were measured. CVR values higher than 0.62 and CVI values higher than 0.79 were accepted. At this stage, five questions were withdrawn from the questionnaire. The reliability of the instrument was measured using the internal consistency method. The questionnaire was completed by 30 MS patients. The Cronbach’s alpha value for the questionnaire was 87. To complete the questionnaires, trained interviewers went there for four consecutive weeks between 23 and 2022 and 17 November 2022. After signing the written consent form, the questionnaires were provided with the objectives and completed under the supervision of the interviewers. Questionnaires were checked by the interviewers after answering by the people, and the incomplete answers were completed by the people with the guidance of the interviewers.

### Data analysis

Data analysis was done in a descriptive way using frequency and percentage indicators for qualitative variables and mean and standard deviation for quantitative variables. To investigate the factors affecting MS patients’ quality of life, multivariable linear regression was implemented to control the influence of confounding variables, so that initially univariate regression was implemented and the variables that considered a significance of less than 0.25 were entered into the multivariable model. Data analysis was done using SPSS version 17 and at a significance level of less than 0.05.

## Result

This study was conducted on 223 people with MS. Most of the subjects were women (72.6%, 162 people), 40.4% were in the early stage of the disease; the majority of people were married (70.9%, 158 people) with the average (standard deviation) age and duration of the disease being 37.8 (8.3) and 8.6 (6.1) years, respectively. The average (standard deviation) score of patients’ overall quality of life was 63.3 (21.3), the highest score in terms of different aspects of quality of life related to emotional role limitations was 76.1 (20.3) and the lowest score related to sexual function satisfaction was 47.5 (20.1) (Table 1).

**Table 1:** Descriptive statistics of research variables

**Variable**		**Frequency (%)**
Gender	Female	162 (72.6%)
	Male	61 (27.4%)
Current disease course	RR*	58 (26%)
	PP**	90(40.4%)
	SP***	75 (32.7%)
Marital Status	single	65 (29.1%)
	married	158 (70.9%)
**Variable**		**Mean (SD)**
Age		37.8 (8.3)
Duration of disease		8.6 (6.1)
Physical health		69.4 (19.9)
Physical role limitations		72.3 (19.6)
Emotional role limitations		76.1(20.3)
Pain		51.8 (19.9)
Emotional well-being		61.6 (12.2)
Energy		57.9 (13.3)
Health Perception		58.1 (15.4)
Social function		54.5 (14.9)
Cognitive functioning		67.2 (20.4)
Health distress		66.9 (22.6)
The overall quality of life		63.3 (21.3)
Sexual function		47.8 (22.7)
Change in health		53.9 (24.4)
Sexual function satisfaction		47.5 (20.1)
Social stigma		46.9 (12.6)

*Relapsing-Remitting- ** Primary progressive- *** Secondary progressive

Based on univariate regression, marriage [B = 0.2, p-value = 0.004], physical health [B = 0.4, p-value < 0.001], emotional well-being [B = 0.4, p-value = 0.001], cognitive functioning [B = 0.4, p-value < 0.001], health distress [B = 0.5, p-value < 0.001] had a positive and significant relationship with patients’ quality of life. Duration of disease [B=-0.2, p-value < 0.001], physical role limitations [B=-0.4, p-value < 0.001], emotional role limitations [B=-0.5, p-value < 0.001], pain [B=-0.4, p-value < 0.001], energy [B=-0.3, p-value = 0.02], health perception [B=-0.3, p-value = 0.001], social functioning [B=-0.4, p-value < 0.001], sexual function [B=-0.3, p-value < 0.001], change in health [B=-0.3, p-value < 0.001], sexual function satisfaction [B=-0.3, p-value < 0.001] and social stigma [B=-0.3, p-value < 0.001] had a negative and significant relationship with patients’ quality of life. (Table 2).

**Table 2:** Correlation coefficients of study variables in univariate analysis model

Variable	Uni variate	
	B coefficient	P value
Gender	-0.4 (0.8, 4.6)	0.6
Age	-0.2 (-0.5, 0.2)	0.3
Current disease course	-11.4 (-15.6, -7.2)	0.3
Marital Status	0.2 (2.9, 15.2)	0.004
Duration of disease	-0.2 (-1.1, -0.2)	< 0.001
Physical health	0.4 (0.3, 0.5)	< 0.001
Physical role limitations	-0.4 (-0.5, -0.2)	< 0.001
Emotional role limitations	-0.5 (-0.5, -0.3)	< 0.001
Pain	-0.4 (-0.5, -0.2)	< 0.001
Emotional well-being	0.4 (0.1, 0.6)	0.001
Energy	-0.3 (-0.5, -0.1)	0.02
Health perception	-0.3 (-0.5, -0.1)	0.001
Social function	-0.4 (-0.6, -0.3)	< 0.001
Cognitive functioning	0.4 (0.3, 0.5)	< 0.001
Health distress	0.5 (0.4, 0.6)	< 0.001
Sexual function	-0.3 (-0.5, -0.2)	< 0.001
Change in health	-0.3 (-0.4, -0.2)	< 0.001
Sexual function satisfaction	-0.3 (-0.4, -0.2)	< 0.001
Social stigma	-0.3 (-0.7, -0.3)	< 0.001

Based on the results of multivariate regression, by controlling the impact of confounding variables, patients’ quality of life with emotional role limitations [B=-0.2 (-0.3, -0.03), p-value = 0.02], energy [B=-0.3 (-0.8, − 0.4), p-value < 0.001], sexual function [B=-0.1 (-0.3, -0.02), p-value < 0.001] had a negative and significant relationship. On the other hand, health distress [B = 0.4 (0.2, 0.5), p-value < 0.001] and cognitive functioning [B = 0.2 (0.005, 0.4), p-value = 0.04] have had a positive and significant relationship with patients’ quality of life (Table 3).

**Table 3:** Correlation coefficients of study variables in multivariate analysis model

Variable	multivariate	
	B coefficient	P value
Emotional role limitations	-0.2 (-0.3, -0.03)	0.02
Energy	-0.3 (-0.8, -0.4)	< 0.001
Cognitive functioning	0.2 (0.005, 0.4)	0.04
Health distress	0.4 (0.2, 0.5)	< 0.001
Sexual function	-0.1 (-0.3, -0.02)	0.03

## Discussion

The results of the study showed people with MS quality of life were positively related to marriage, physical health, emotional well-being, cognitive functioning, and health distress; and negatively related to the duration of disease, physical role limitations, emotional role limitations, pain, energy, health perception, social functioning, sexual function and change in health. The most common type of disease course is PP. The majority of people with MS covered by the Jahrom MS Association were women.

People with MS’s quality of life were positively related to marriage, physical health, emotional well-being, cognitive functioning and health distress. Several studies have emphasized the relationship between the extent and severity of symptoms and disorders with the quality of life of people with MS [22–26]. The results of Fernández-Muñoz’s study showed a negative correlation between emotional well-being and dissatisfaction with life in patients [27]. On the other hand, there was a negative correlation between the duration of disease, physical role limitations, emotional role limitations, pain, energy, health perception, social functioning, sexual function and change in health with patients’ quality of life. There was also a negative relationship between social stigma and patients’ quality of life. According to the findings of the present study, pain and cognitive disorders are known as areas with a negative relationship with patients’ quality of life [28–30]. Also, according to Fernández’s study, cognitive disorders harmed the quality of life of people with MS [31]. On the other hand, the results of studies have shown that social isolation and wishful thinking, behavioral non-participation, distancing and distraction are related to lower quality of life in MS patients [32–35]. The results of Kalantari [13] and Strober [36] studies showed that because of social stigma, many people with MS prefer to hide their disease to maintain a job, maintain social relationships, and fear the reaction and behavior of others in society. It seems that paying attention to personality and psychological factors can cause an important effect in improving the quality of life of MS patients, and it is necessary to improve the self-esteem and self-efficacy of these patients.

The results of the study showed the most common type of disease course was PP; consistent with these results, the study by Sahraian showed a higher prevalence of PP [23], while in Rezaali’s study, most of the patients suffered from RR symptoms [22]. Approximately 85% of people with MS are initially diagnosed with RR. This stage generally enters the progressive stages after 8 to 20 [27].

In the present study, age had a weak negative relationship with people with MS quality of life; as in Calandr’s study, age was identified as a risk factor for the quality of life in people with MS [37].

In this study, the demographic characteristics, the domains related to the quality of life and the relation between social stigma and the quality of life of people with MS were investigated. The majority of people with MS covered by the Jahrom MS Association were women. Rezaali’s study in Qom (a central city in Iran) indicated the prevalence of MS in women more than three times that of men [38]. Also, the results presented by Sahraian have shown there are more women among Iranian people with MS [39]. According to Magyari’s study, the risk of MS in Danish women is doubled compared to men [40]. The findings of Magyari’s review study also support the predominance of women in the incidence and prevalence of MS [41]. These results are consistent with the findings of the present study. Hormonal factors can play a role in this gender difference [42]. On the other hand, it seems that pregnancy causes a protective effect against MS, and the decrease in pregnancies in recent decades has helped to increase the gender ratio and incidence of MS in women [40]. Encouraging childbearing in countries like Iran, where the population is aging, can carry out an important role in reducing the prevalence of MS in women, in addition to helping to reduce demographic problems.

It is suggested that future studies investigate the impact of individual education and social interventions on reducing social stigma and improving various aspects of the quality of life of people with MS.

One of the strengths of the study was the presence of the majority of people with MS from Jahrom City in the study. Also, answering the questions was done under the supervision of the interviewer and accurately. The limitations of the research included the difficulty in obtaining the opinions of the participants due to the physical problems of some participants due to the severity of the disease. The large number of questions in the questionnaires remain another problem of this study; to solve this problem, after completing half of the questions, the participants received a short break and were treated. If necessary, the interviewer reads the question and directly transfers the answers of the people to the questionnaire. One of the other problems was the failure of some people to go to the place of the MS Association to answer the questionnaires, which we tried to solve by calling and inviting them. On the other hand, the Covid-19 pandemic and its dangers for people with MS made it difficult to reach the contacts in person.

## Conclusion

The results of the study showed factors such as marriage, physical health and health distress had a positive relationship and factors such as duration of disease, physical role limitations, emotional role limitations, pain, energy, health perception and social stigma have a negative relationship with the quality of life of people with MS. The quality of life of people with MS is more influenced by mental and psychological factors than the physical limitations of these patients. The quality of life in people with MS should be taken into consideration by health personnel due to the impact it has on the adaptation and coping strategies of this disease. It is also very important to pay attention to the solutions to deal with social stigma as a disruptive factor in the quality of life of these people. To improve the quality of life in these patients and reduce the impact of social stigma on their quality of life, more emphasis should be placed on providing individual or group psychological counseling for these patients. Also, programs should be implemented to improve public knowledge and teach how to deal with these patients in society.

## Data Availability

All data generated or analyzed during this study are included in this published article.
